# Efficacy of Zinc-, Copper-, and Silver-Doped Fluorapatite as Bacteriophobic Surfaces for Percutaneous Osseointegrated Device Applications

**DOI:** 10.1002/cnma.202500276

**Published:** 2026-03-29

**Authors:** Sujee Jeyapalina, Samantha K. Steyl, Pooya Elahi, Jill Shea, Ruben Sundramurti, Jayant Agarwal, James Peter Beck

**Affiliations:** 1 Plastic and Orthopaedic Surgery Research Laboratory, George E. Wahlen Department of Veterans Affairs Medical Center, Salt Lake City, Utah, USA; 2 Division of Plastic Surgery, Department of Surgery, University of Utah School of Medicine, Salt Lake City, Utah, USA; 3 Department of Biomedical Engineering, University of Utah, Salt Lake City, Utah, USA; 4 Department of Orthopaedics, University of Utah School of Medicine, Salt Lake City, Utah, USA

**Keywords:** antimicrobial metal doping, bacteriophobic surfaces, characterization, keratinocyte adhesion, metal-doped fluorapatite

## Abstract

Percutaneous osseointegrated (OI) prosthetic systems offer superior exoprosthetic docking compared with traditional socket-based attachments; however, their clinical success remains limited by high superficial infection rates. These infections arise primarily from the absence of a stable epithelial seal at the implant–skin interface, permitting bacterial colonization of the stoma, particularly by *Staphylococcus aureus* (*S. aureus*). Fluorapatite (FAp) coatings have previously demonstrated potential to enhance epithelial integration, yet additional antimicrobial functionality could limit bacterial colonization on the device. This study investigated metal doping as a strategy to create dual-functional FAp surfaces that resist bacterial adhesion for percutaneous OI applications. Zinc-, copper-, and silver-doped FAp were synthesized in-house using a validated precipitation method and comprehensively characterized. Sintered discs were evaluated for *S. aureus* adhesion and cytocompatibility to keratinocytes. Fourier transform infrared confirmed retention of the hexagonal apatite structure across all doping compositions. X-ray diffraction revealed secondary phases at higher dopant levels. SEM showed dopant-dependent microstructural changes. Only copper-doped FAp (1%–5%) reduced *S. aureus* adhesion by >3 log-folds. However, copper-doped surfaces exhibited reduced cytocompatibility to keratinocytes, highlighting a tradeoff between anti-bacterial adhesion efficacy and epithelial attachment affinities. These findings indicate that lower copper doping levels warrant further investigation to achieve keratinocytes adhesion for percutaneous implant coating applications.

## 1 | Introduction

Percutaneous osseointegrated (OI) devices have been developed as alternatives to socket-suspension prostheses [1–7]. In this system, an osseointegrating endoprosthesis is inserted into the medullary canal of the residual long bone, and a bridging connector protrudes through the skin barrier (stoma) to provide a stable platform for exoprosthesis attachment. Four main percutaneous OI prosthetic systems are used worldwide: 1) OI Prostheses for the Rehabilitation of Amputees (OPRA), 2) Percutaneous OI Prosthesis (POP), 3) the Zimmer Biomet Compress, and 4) German Endo-Exo/Australian OI Percutaneous Leg (OPL) [8]. Although these percutaneous OI devices are used clinically elsewhere, their adoption in the USA has been limited due to the lack of FDA approvals. To date, only OPRA has been FDA-cleared for clinical use in the United States, while the FDA has approved POP for a clinical trial. However, many US surgeons are now implanting the other two systems under custom protocols. Regardless of implant design, all current systems face challenges related to infection, with reported rates varying widely from 0% to 77%, depending on the implant type and surgical technique employed [5, 9–19], and an overall incidence of ~16% infection across all patients [20]. Consequently, the long-term success of percutaneous implant systems is constrained by the risk of infection originating at the implant exit site/stoma.

Reported high infection rates are attributed to the absence of an epithelial seal around the implant surface. It has been proposed that the reepithelialization phase of wound healing is disrupted by the presence of skin-penetrating implants, preventing the complete restoration of epidermal continuity [21–23]. As a result, the epithelial tissue may migrate proximally along the implant surface in an attempt to reestablish the barrier, which results in a sinus tract around the implant post. This space can serve as an immune-privileged *niche*, providing a protected environment for bacterial colonization. The resulting absence of a skin seal allows potential pathogens to reach the underlying soft tissue and results in the host’s immune response.

It is known that bacteria colonize all stomas (implant exit sites) of percutaneous OI devices [24, 25]. While some of these bacteria are potentially pathogenic, the majority of the stoma microbiota are commensal, forming a stable microbial community that can help prevent pathogenic overgrowth. In a mature, stable stoma—representing an optimally healed wound—these commensal populations may act as mutualistic partners, contributing to the maintenance of local tissue homeostasis and limiting the risk of clinical infection. Nevertheless, these systems are associated with high infection rates, with *Staphylococcus aureus* (*S. aureus*) being the most frequently identified pathogen [11, 12, 25–27]. Infection is believed to occur when the microbial balance shifts, allowing pathogenic species to dominate, highlighting the need for strategies to protect the interface. Additionally, protection during the initial healing period also becomes more important.

One strategy proposed to reduce infection rates around percutaneous implants is to enhance the integration of dermal and epidermal tissues with the implant surface. Numerous studies have attempted to establish a durable skin-to-implant seal; however, a long-term, clinically reliable solution has yet to be realized [28–41]. Building on the natural model of the dental enamel–junctional epithelium interface, our group previously demonstrated that fluorapatite (FAp) coatings on implant surfaces can improve epithelial healing responses. Specifically, FAp promotes epithelial cell differentiation, reduces downgrowth, and decreases the extent of granulation tissue formation at the stoma [36, 39]. Despite these benefits, as stated above, the implant exit site may remain susceptible to bacterial invasion during the wound-healing period. Although routine stomal hygiene with mild soap and water reduces bioburden on the exposed implant surface, further surface anti-bacterial adhesion protection would be advantageous. Ideally, the percutaneous component itself would provide an additional antimicrobial barrier to mitigate early-stage infection risk.

It is well established that zinc (Zn), copper (Cu), and silver (Ag) ions possess antimicrobial properties, and incorporation of these ions into hydroxyapatite (HAp) through ionic substitution of calcium (Ca) has been shown to enhance the antimicrobial activities [42–54]. Despite the close structural and chemical similarities between HAp and FAp, relatively few studies have examined the antimicrobial potential of these metal-doped FAp formulations [55–57]. The present study, therefore, sought to further evaluate the bacterial adhesion properties of Zn-, Cu-, and Ag-doped FAps surfaces to be used as coatings for percutaneous OI implants. Based on prior findings demonstrating the antimicrobial effects of metal-doped HAp, we hypothesized that incorporating small molar percentages of Zn, Cu, or Ag ions into the FAp crystal structure would enhance its bacteriophobic properties while maintaining its compatibility with keratinocyte adhesion and differentiation. To test this hypothesis, metal-doped FAp powders were synthesized using 1%–10% molar substitution of Ca ions, and their ability to resist bacterial adhesion was assessed against *S. aureus*. Finally, cytocompatibility of the doped FAp materials was evaluated using human keratinocyte cells. A positive outcome would support the development of a dual-function FAp-based coating that both promotes wound healing and provides less bioburden at the implant exit sites, thereby potentially reducing infection risks associated with percutaneous OI devices.

## 2 | Materials and Methods

### 2.1 | Chemicals

Unless otherwise specified, all reagent-grade chemicals were purchased from Sigma Aldrich (St. Louis, MO).

### 2.2 | FAp and Doped-FAp Synthesis and Characterization

Pure FAp powder was synthesized employing a well-established precipitation technique [58]. In short, a 2.4 M calcium nitrate tetrahydrate (Ca(NO_3_)_2_.(H_2_O)_4_) and a mixed solution of 1.44 M sodium phosphate dibasic (Na_2_H(PO_4_)) and 0.54 M sodium fluoride (NaF) were gradually introduced at a rate of 2.8 mL/min to a 20 L round-bottom flask containing 10 L of boiling water. The pH of the reaction mixture was rigorously maintained at 9.2 for the entirety by titrating the reaction mixture with 2 M sodium hydroxide (NaOH) using an auto-titrator (Radiometer Analytical, Hach Company, Loveland, CO). Following synthesis, the mixture was stirred for 1 h and left to complete the reaction overnight. The resulting insoluble FAp powder was thoroughly washed with 20 L of deionized water to eliminate all soluble residual salts. It was subsequently collected and dried in an oven at 60°C for 48 h to remove excessive water from the product.

As shown in Table 1, precise concentrations of calcium nitrate and the respective metal nitrates were calculated to synthesize metal-doped FAp at the predetermined substitution levels (1, 2, 5, and 10 mol%), where nitrate ratios were calculated to maintain the characteristic metal-to-phosphate ratio of the apatite structure (1.67). Zinc nitrate hexahydrate (Zn(NO_3_)_2_·6H_2_O), copper nitrate trihydrate (Cu(NO_3_)_2_·3H_2_O), and silver nitrate (AgNO_3_) served as the sources of Zn, Cu, and Ag, respectively. The synthesis procedures followed those described for undoped FAp. The resulting powders were designated as 1, 2, 5, and 10ZnFAp; 1, 2, 5, and 10CuFAp; and 1, 2, 5, and 10AgFAp, and were subsequently subjected to characterization. All characterization was performed on as-synthesized powders without heat treatment or sintering, except for X-rRay diffraction (XRD) analysis, which was conducted on powders calcined at 900°C to reduce peak broadening and confirm crystalline phases.

### 2.3 | Inductively Coupled Plasma Mass Spectrometric (ICP-MS) Analysis

ICP-MS (Thermo Scientific, Waltham, MA) was used to quantify Ca, phosphate, Zn, Cu, and Ag relative weight in the synthesized powders. For this purpose, a known weight of each sample was initially digested using ultrapure, spectroscopic-grade nitric acid. An internal standard and a blank were used for calibration. The prepared samples were then introduced (nebulized) into the inductively coupled plasma, where the atoms were atomized and ionized. The resulting ions were separated by their mass-to-charge ratio in the mass analyzer, detected, and recorded. Elemental concentrations were calculated and expressed as mg/kg, and the molar ratios of Ca to each dopant (Zn:Ca, Cu:Ca, Ag:Ca), as well as the combined (Ca + dopant):phosphate ratio, were subsequently calculated.

### 2.4 | Fourier Transform Infrared (FTIR) Spectroscopic Analysis

FTIR spectra of the doped and undoped FAp powders, as synthesized, were obtained by mixing the powders with spectroscopic-grade potassium bromide (KBr) and pressing them to make discs. Briefly, 0.003 g of apatite powder and 0.027 g of KBr were finely ground to minimize scattering effects and absorption distortions. The mixture was then compressed using 440C stainless-steel dies fitted with a hydraulic press (Specac; Fort Washington, PA). The total weight of apatite powder was maintained at 0.3 wt% of KBr to comply with the Beer–Lambert law [59, 60]. The resulting discs were characterized using an FTIR spectrometer (Thermo Fisher Scientific Nicolet iS50; Waltham, MA) equipped with a DTGS detector, recording transmittance (%) over the wavenumber range of 400–4000 cm^−1^ under a purged N_2_ atmosphere.

### 2.5 | Particle Size Analysis (PSA)

After preparing colloidal suspensions of the as-synthesized metal-doped FAp powders in deionized water, the particle size distributions were determined using Dynamic Light Scattering (DLS; Wyatt DynaPro NanoStar, Santa Barbara, CA).

### 2.6 | XRD Studies

The XRD diffractograms of all synthesized metal-doped FAp powders, calcined at 900°C in a furnace (Sentro Tech; Strongsville, OH), were obtained. Ni-filtered Cu-Kα_1_ monochromatic radiation (λ = 1.5418 Å) was used in a 2θ scanning range of 10°–80°, with a step size of 0.02° and a counting time of 1 s per step, using an X-ray diffractometer (Bruker D8 Discover; Billerica, MA). Phase identification and quantitative phase analysis were conducted. Data smoothing and Kα_2_-stripping were applied to achieve the best fit, following the methods of Savitzky–Golay and Rachinger [61, 62]. Full-pattern Rietveld refinement was performed using Bruker DIFFRAC.EVA software (Billerica, MA). For phase identification and crystallinity determination, the XRD patterns were matched to crystallography open database (COD) references with file numbers 1010996, 9008877, 9014580, 1100136, and 2300449, corresponding to FAp, zinc oxide (ZnO), copper oxide (CuO), metallic silver (Ag), and fluorite (CaF_2_), respectively.

### 2.7 | Scanning Electron Microscopic (SEM) Studies

To visualize surface topographies of the discs intended for in vitro assays, compressed discs were prepared and sintered at 1150°C. Briefly, 0.3 g of the respective metal-doped FAp powders was weighed and mixed with 65 μL of sterile water. The mixture was finely ground using a mortar and pestle, die-pressed (Specac; Fort Washington, PA), and sintered at 1150°C for 2 h at a heating/cooling rate of 5°C/min. Surface morphology was examined using a SEM (FEI Quanta 600F; Hillsboro, OR) equipped with energy-dispersive X-ray spectroscopy (EDS; EDAX LLC, Pleasanton, CA). Samples were imaged under high vacuum with a 20 kV electron beam and a spot size of 6.0 to obtain publication-quality images.

### 2.8 | Determination of Bacterial Adhesion Properties

To assess the bacteriostatic properties of doped FAp powders (ZnFAp, CuFAp, and AgFAp), undoped FAp (control), and Titanium (Ti, control, surfaces that are currently used for fabricating percutaneous OI devices), sintered discs were exposed to a known number of *S. aureus* (ATCC 25923). The rationale for using *S. aureus* for this study was, as stated in the introduction, that it is the most frequently identified pathogen in percutaneous OI infections. Prior to bacterial exposure, discs were fabricated as described above. The discs were then sintered at 1150°C for 2 h (Sentro Tech; Strongsville, OH), cleaned with 70% ethanol, and sterilized by steam.

Fresh frozen isolates of *S. aureus were* recovered from stock, streaked onto sterile Tryptic Soy Agar (TSA) plates, and incubated overnight. Ten single colonies were picked and inoculated into 100 mL of sterile Tryptic Soy Broth (TSB; 30 g/L), then incubated for 6–8 h at 36°C. Bacteria were washed three times with 50 mL sterile PBS by centrifugation and resuspension and finally resuspended in 10 mL PBS. Bacterial concentrations were determined using a 1:10 serial dilution, and the suspension was adjusted to 1×10^6^ cells/mL with sterile PBS for inoculation.

For the bacterial adhesion study, four discs for each material type were placed in individual wells of sterile 12-well plates, and 50 μL of the bacterial suspension (1 × 10^6^ CFU/mL) was placed carefully on the surface of each disc. The plates were then transferred to the incubator with 100% humidity and incubated at 37°C for 2 h without disturbing the bacterial drop. After incubation, the discs were washed with sterile PBS to remove unattached bacteria and placed in 1 mL PBS. Adhered cells were detached from the surface by vortexing each sample for 30 s, sonicating for 5 min, and then vortexing for an additional 30 s. The number of adhered bacteria was quantified using the serial dilution method and reported as colony-forming units (CFU) per unit area.

### 2.9 | Cytocompatibility to Keratinocyte Adhesions

To determine doped surfaces’ ability to promote cell adhesion, proliferation, and differentiation, human keratinocytes—the relevant cell type for evaluating FAps as percutaneous device coating materials—were used. Discs were fabricated and sintered as described in the bacterial adhesion study section. For this assay, human epidermal keratinocytes (HaCaT; passages 5–7; T0020001, AddexBio Technologies, San Diego, CA) were seeded at a density of 6,000 cells/cm^2^ and cultured in nondifferentiating growth medium (DMEM + 10% FBS) for predetermined time points of 3, 7, and 12 days. Experiments were carried out for 12-weeks for two reasons: 1) the dividing rates of keratinocytes at low density are very long and 2) to confirm the surfaces’ ability to support the adhered cells.

To facilitate cell adhesion, the cell suspension was carefully pipetted onto each disc and allowed to adhere for 2 h before adding additional culture medium. The medium was refreshed every 2–3 days. At days 3, 7, and 12, *n* = 4 discs /surface/time point were removed, rinsed with PBS, and transferred to new well plates. Three milliliters of culture medium containing 10% alamarBlue (Thermo Scientific; Waltham, MA) was added to each well. After 2 h of incubation, the metabolic activity of adhered cells was measured at 560–590 nm using a SpectraMax M3 plate reader (Molecular Devices; Sunnyvale, CA). A calibration curve was generated using known numbers of HaCaT cells in 3 mL of 10% AlamarBlue, incubated for 2 h. This curve was used to calculate cell counts from the measured fluorescence values. Data are reported as the number of adhered cells per unit area (cm^2^) of the disc surface.

### 2.10 | Statistical Analysis

Unless otherwise specified, the data are presented as means ± standard error of the mean (SEM). The significance of the bacterial adhesion and cytocompatibility data was initially assessed using one-way analysis of variance (ANOVA). Following a statistically significant result from the ANOVA, Tukey’s honest significant difference (HSD) test was employed for pairwise comparisons. The predetermined significance level was set at *p*<0.05. All statistical analyses were performed using Stata Statistical Software: Release 18 (StataCorp LLC, College Station, TX).

## 3 | Results

The in-house synthesized materials were characterized using ICP-MS, FTIR, PSA, and XRD. Table 2 presents the measured dopant/Ca and (dopant + Ca)/phosphate molar ratios for all synthesized metal-doped apatites, calculated from ICP-MS data. These results revealed that Ca^2+^ ions were successfully substituted with approximately 1%, 2%, 6%–8%, and 11%–19% of the selected dopant metal ions, while the (dopant + Ca)/phosphate molar ratios ranged from 1.56 to 1.93.

**FIGURE 1 |:**
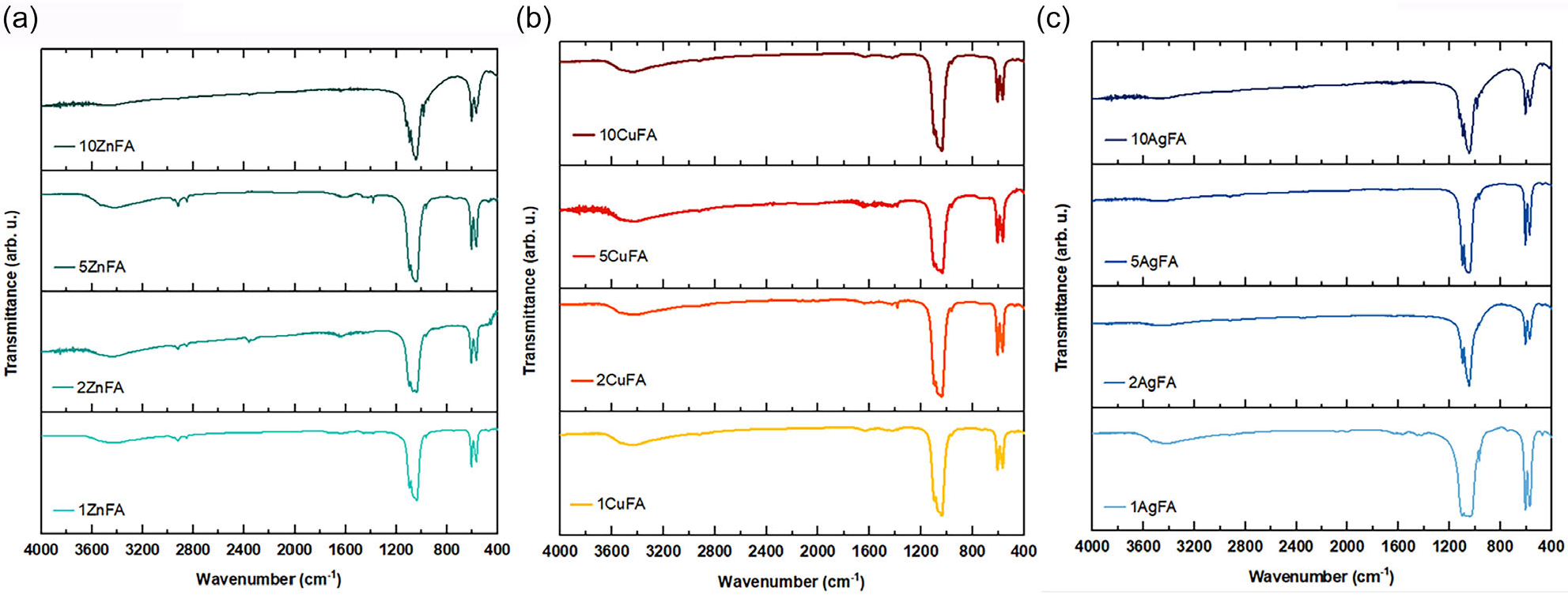
A representative set of FTIR spectra of metal-doped FAp samples, highlighting the characteristic phosphate peaks of the apatite molecular structure. (a) Zn-doped, (b) Cu-doped, and (c) Ag-doped FAps.

A representative set of FTIR spectra of unsintered metal-doped FAp powders is given in Figure 1. Data reveals that all metal-doped FAp samples displayed characteristic phosphate peaks (PO_4_^3−^): ν_3_ asymmetric stretching vibration of PO_4_^3−^ at ~1090 cm^−1^ and ν_4_ O–P–O bending vibrations at ~570–600 cm^−1^. The absence of a sharp peak at 3565 cm^−1^ confirmed that all hydroxyl groups were substituted by the fluoride ions, indicating successful synthesis of the intended FAp structure. Additionally, carbonate (CO_3_^2−^) peaks were observed at ~880, 1070, and 1480 cm^−1^.

**FIGURE 2 |:**
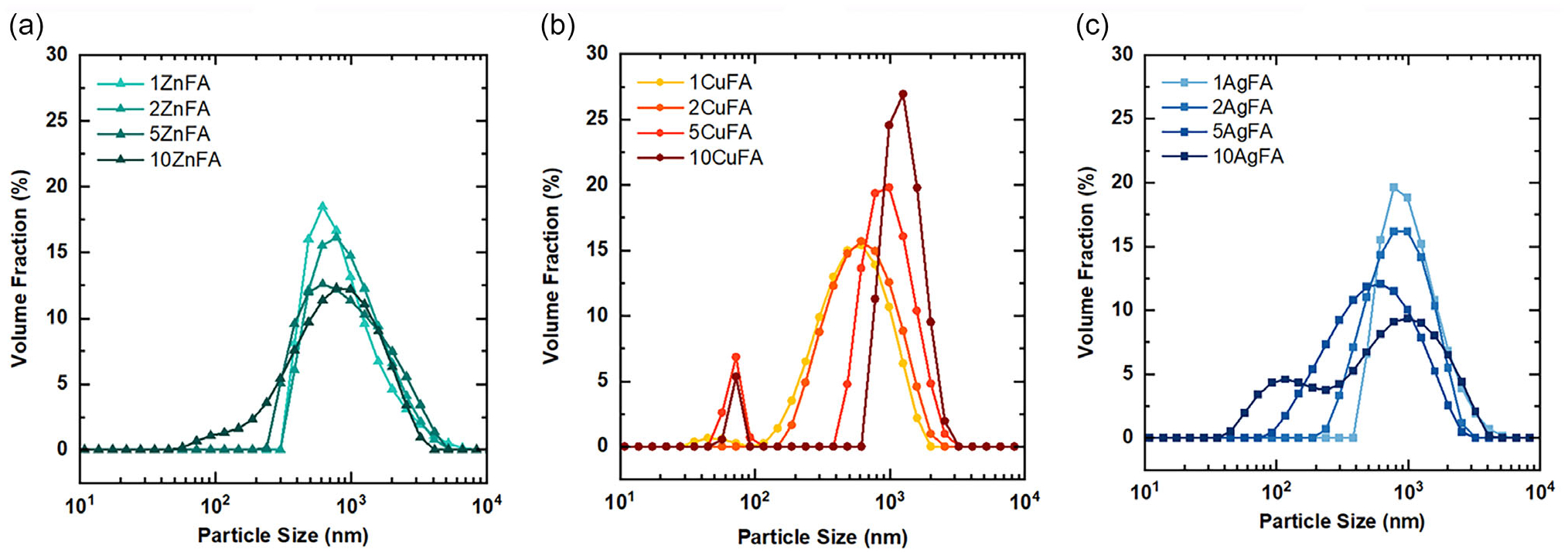
Measured particle size distributions of the studied apatites. These scattergrams show volume fraction (%) as a function of particle size (nm) for all 12 metal-doped FAp samples. (a) Zn-doped, (b) Cu-doped, and (c) Ag-doped FAps.

Figure 2 presents the particle size distributions for each metal-doped FAp. All doped FAp samples exhibited unimodal distributions when doped with 1%–2% respective metals; however, both the distribution width and peak particle size varied with dopant type and percentage. ZnFAp exhibited Gaussian-like distributions at all doping levels studied, with peak particle sizes of approximately 800–1000 nm (Figure 2a). In contrast, 1CuFAp and 2CuFAp showed unimodal distributions centered around 100–300 nm, whereas 5CuFAp and 10CuFAp displayed bimodal distributions (Figure 2b). At 5% Cu substitution, the distribution broadened and became bimodal, with the central peak shifting to larger particle sizes (~300–500 nm). The 10CuFAp sample exhibited a distinct sharp peak at approximately 800 nm. AgFAp (1–5AgFAp) displayed Gaussian-like distributions similar to ZnFAp, with peak particle sizes ranging from ~500 to 800 nm, followed by a bimodal distribution of 10AgFAp powders (Figure 2c).

**FIGURE 3 |:**
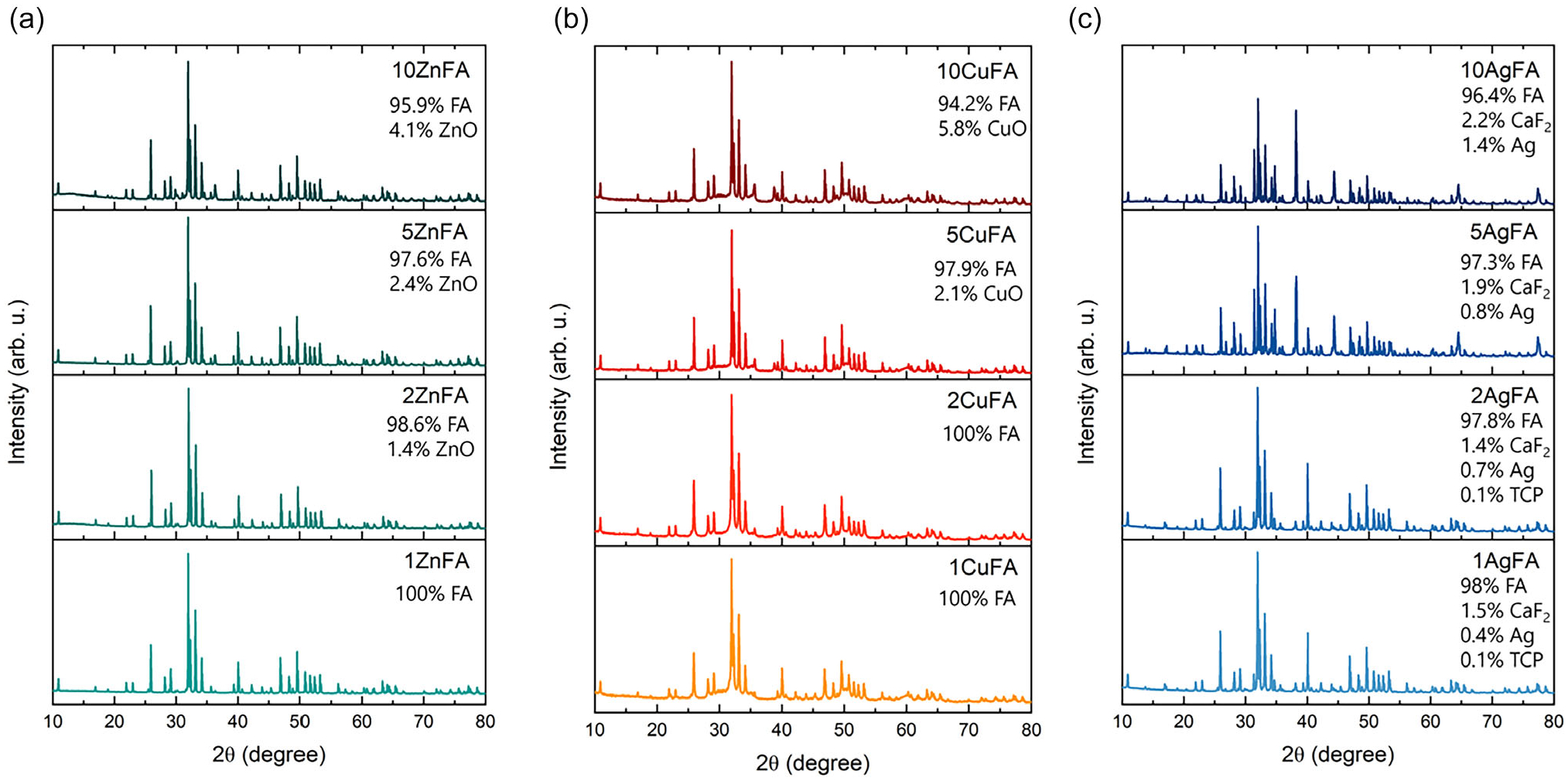
A representative set of diffractograms showing the XRD patterns of all synthesized metal-doped FAps: (a) 1, 2, 5, and 10ZnFAp; (b) 1, 2, 5, and 10CuFAp; and (c) 1, 2, 5, and 10AgFAp.

The XRD diffractograms with quantitative phase identification for all studied metal-doped FAp samples are presented in Figure 3. As explained in the Methods section, these patterns were obtained after calcination at 900°C for 1 h. Patterns may therefore also include temperature-related changes. The XRD results confirmed complete incorporation of the dopant metals into the apatite structure at a 1% doping level for Zn and at both the 1% and 2% levels for Cu, with all three samples exhibiting a single-phase apatite structure. It appears that with increasing Zn doping (2%–10%), up to 5% additional secondary diffraction peaks corresponding to ZnO were observed. For 5% and 10% Cu doping, additional oxide peaks were also visible with ~6% of oxides in the 10CuFAp sample. In contrast, AgFAp displayed mixed phases—including CaF_2_, metallic Ag, and tricalcium phosphate (TCP) byproducts—at all doping levels (Figure 3c).

**FIGURE 4 |:**
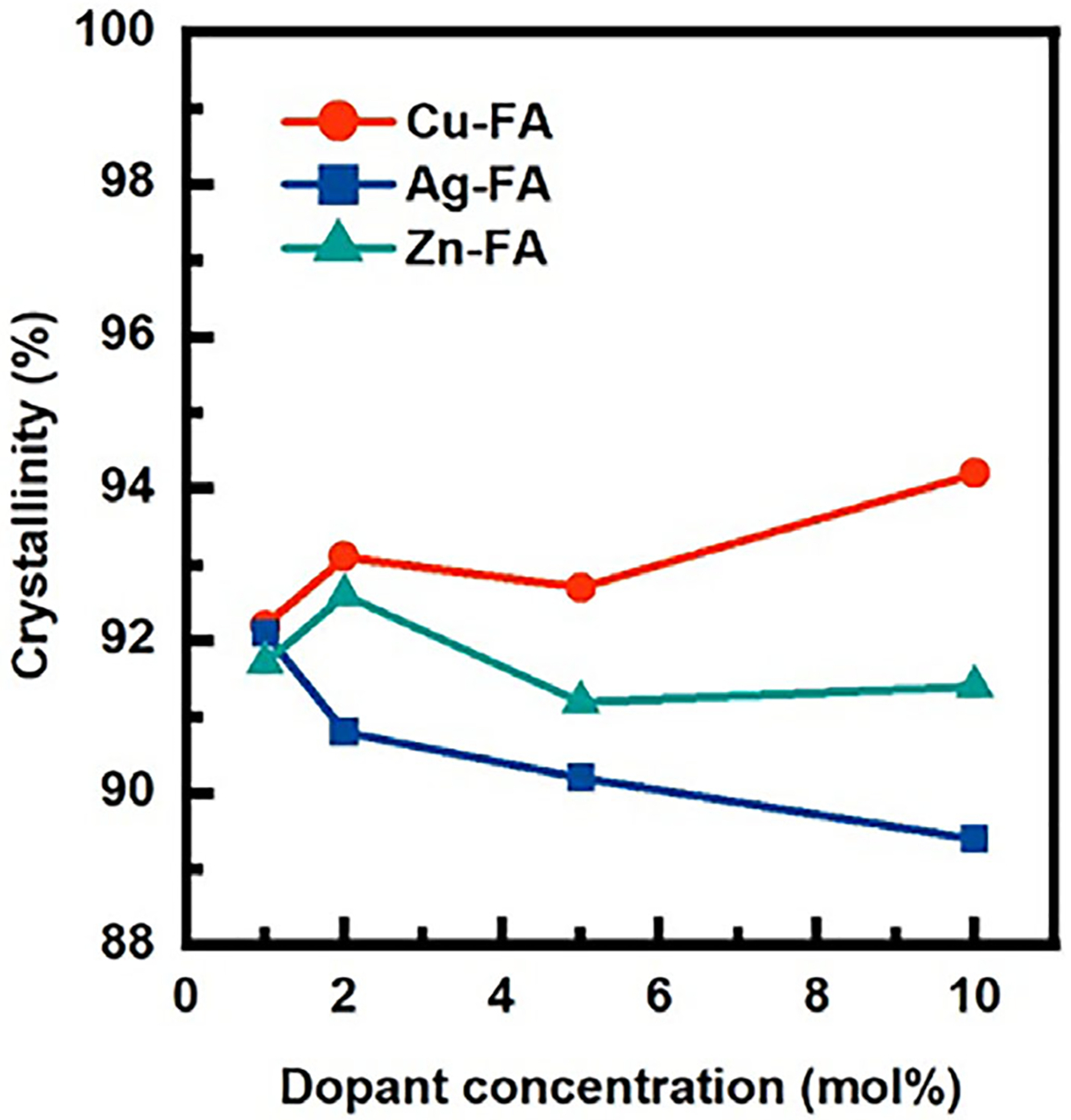
A set of scatter plots showing crystallinity as a function of increasing metal doping. Zn—green, Cu—red, and Ag—blue).

The lattice parameters for each apatite were compared with the reported values for FAp (*a* = 9.375 Å and *c* = 6.887 Å [63]), which are detailed in Table 3. For ZnFAp and CuFAp, the ***a*** and ***c*** parameters decreased slightly with increasing amounts of dopants. Conversely, both the ***a*** and ***c*** lattice parameters for AgFAp leaned to increase with increasing dopant concentration. Based on the XRD data, the overall crystallinity of each metal-doped FAp was also calculated and is presented in Figure 4. All studied metal-doped apatites exhibited high crystallinity (>89%). Crystallinity remained approximately 91%–92% with 1%–10% Zn doping, while increasing Cu doping slightly increased crystallinity. Interestingly, increasing Ag doping led to a decrease in crystallinity.

**FIGURE 5 |:**
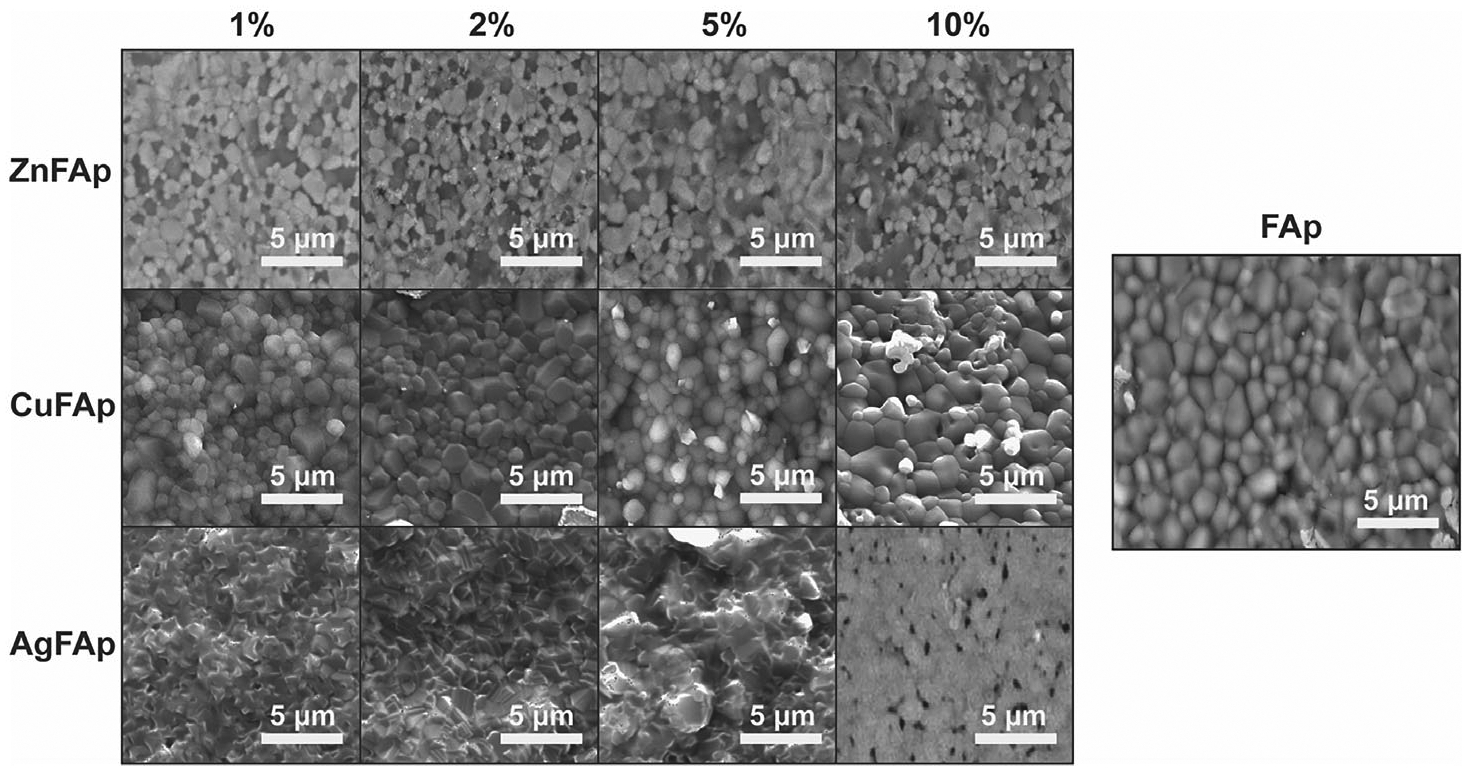
A representative set of scanning electron micrographs showing the surface microfeatures of metal-doped fluorapatites (ZnFAps, CuFAps, and AgFAps) in comparison with undoped FAp. The images highlight differences in particle morphology and surface texture across different dopants and doping levels. Scale bar = 5 μm.

Figure 5 presents SEM micrographs of the sintered discs at 1150°C prior to biological assessments, revealing the surface microtopographies of all samples. For ZnFAp and CuFAp, an increase in grain size was observed with increasing doping from 1% to 10%. In contrast, the surface topographies of 1AgFAp, 2AgFAp, and 5AgFAp did not show the same trend. 10AgFAp, however, exhibited noticeably larger grains, with approximately 10–15 μm in diameter and interposing microporosities. It is worth noting that AgFAps have distinct surface morphologies compared to others surfaces tested in this study.

**FIGURE 6 |:**
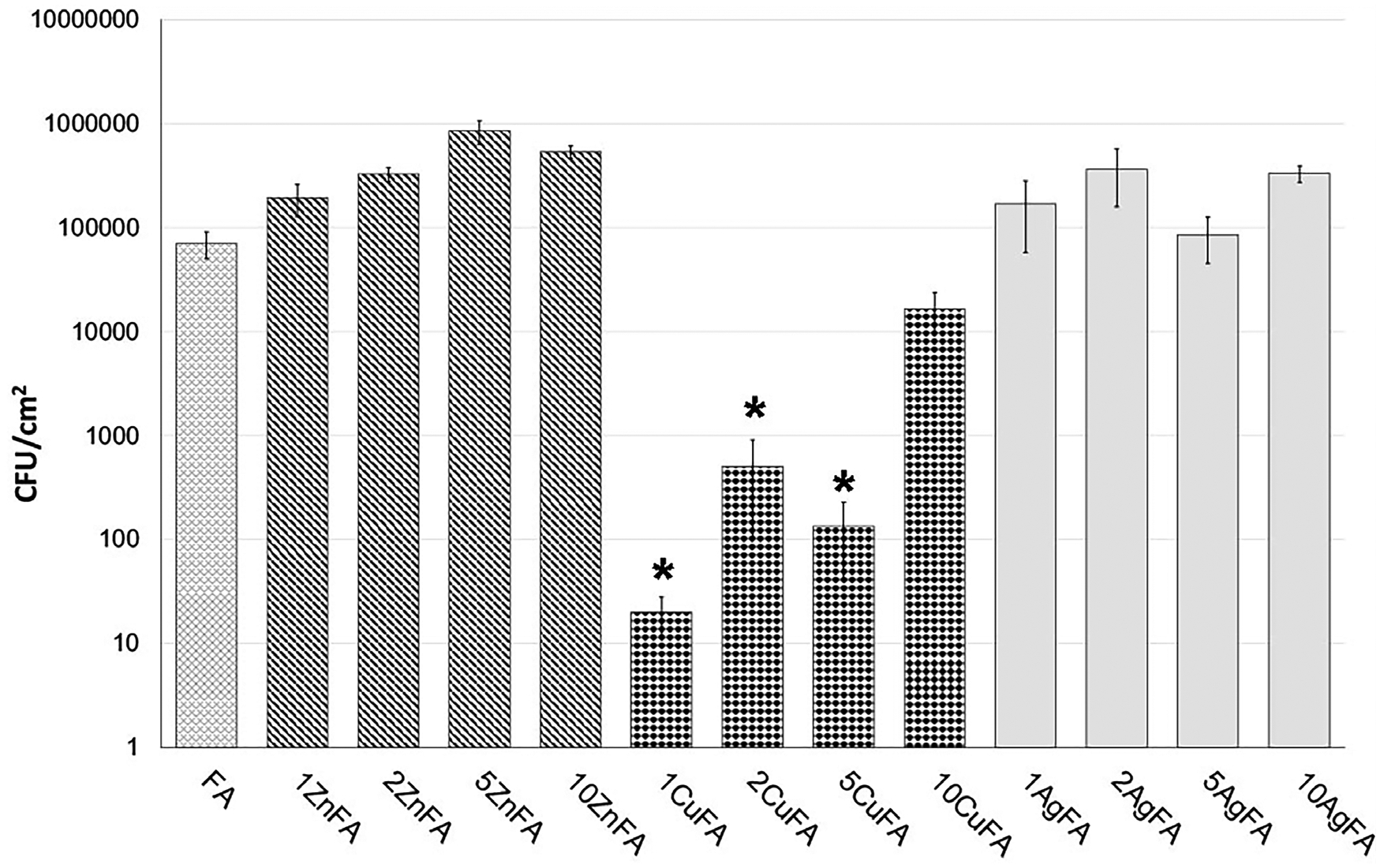
A bar chart showing the number of adhered bacterial (log_10_(CFU/cm^2^)) on Ti, FAp, and metal-doped FAp discs (1–10ZnFAp, 1–10CuFAp, 1–10AgFAp) after 2 h of incubation. Asterisks (*) indicate a significant reduction (*p* < 0.05) compared to all other groups.

To evaluate the surface bacterial adhesion properties of each material, sintered disks of all metal-doped FAp samples were tested using *S. aureus* as the inoculum. After exposure to a known bacterial concentration, the number of adherent bacteria on each surface was quantified following a 2-h incubation, and the data are shown in Figure 6. The results demonstrated over a 3-log reduction in bacterial adhesion with 1%–5% Cu doping. Statistical analysis confirmed that these copper-doped surfaces had significantly fewer adherent bacteria compared to all other tested surfaces. No other significant differences in bacterial adhesion were observed among the remaining groups.

**FIGURE 7 |:**
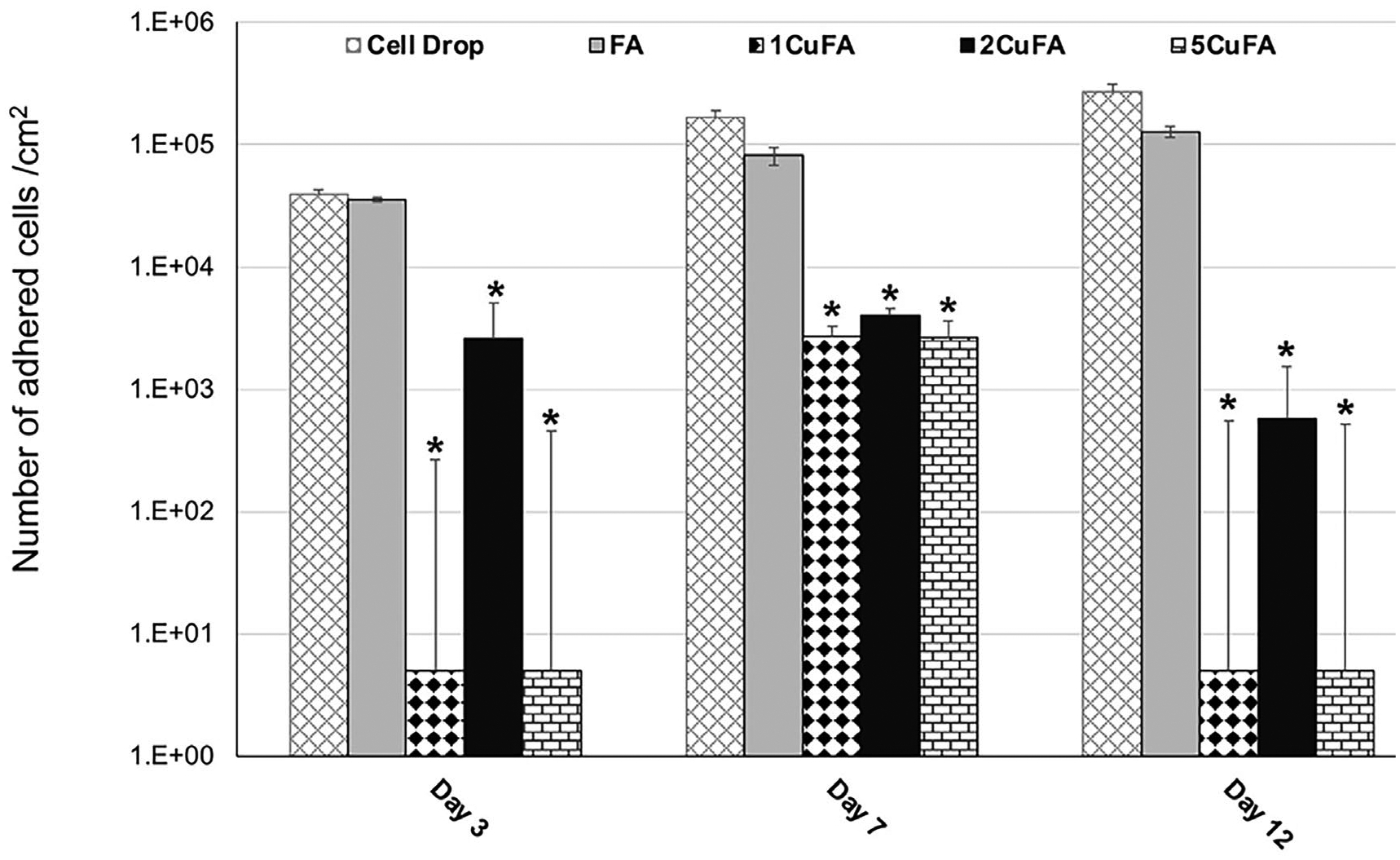
Bar chart showing HaCaT cell counts in cell-only wells and on titanium, FAp, and 1–5CuFAp discs at 3, 7, and 12 days. Asterisks (*) indicate a significant reduction (*p*<0.05) in cell numbers on 1CuFAp, 2CuFAp, and 5CuFAp compared with cell-only and FAp controls at each time point.

The cytocompatibility to keratinocyte adhesion and survival was performed on the metal-doped FAp samples that showed reductions in bacterial adhesion (1CuFAp, 2CuFAp, and 5CuFAp). These samples were evaluated using HaCaT cells over 3, 7, and 12 days. The results are presented in Figure 7. All three copper-doped samples exhibited a significant reduction in keratinocyte adhesion compared to the three controls—cell-only wells, titanium coupons, and undoped FAp discs—indicating reduced cytocompatibility. It appeared that adhered keratinocytes were viable upto 7 days on the doped surfaces.

## 4 | Discussion

This study was designed to test the hypothesis that small molar percentages of Zn, Cu, or Ag incorporated into the crystal structure of FAp would resist bacterial adhesion on the surface while supporting cell growth. The data generated in this study only partially supported this hypothesis, which showed copper doping at 1%–5% resulted in an approximately 3-log fold reduction in *S. aureus* adhesion (*p*<0.05; Figure 6). A 3-log reduction in bacterial adhesion, equivalent to a 99.9% decrease in bacterial load, is generally considered highly effective because it could disrupt early surface colonization by the potential pathogen, *S. aureus*. Additionally, these findings are consistent with previously reported data for Cu-doped HAp [64, 65]. However, these Cu doping levels appeared not to be cytocompatible with keratinocytes’ adhesion. Since there were no significant differences in bacterial adhesion with 1%–5% Cu doping, it is likely that even lower Cu doping levels could maintain bacteriophobic properties while being conducive to cell adhesion and proliferation, suggesting the need for further studies.

The rationale for selecting the doping metals is based on the known antimicrobial properties of elemental Zn, Cu, and Ag. Also, the literature confirms their ability to incorporate into and substitute for Ca^2+^ within the apatite crystal structure during synthesis [66–72]. In addition, these metals are known to exhibit antimicrobial activity in their ionic forms in solution (Table 4). Even though CuO could be considered an impurity, it also has known antimicrobial effects [75, 79, 81]. Based on the values in Table 4, the amounts of metal used in the synthesis of these doped FAps were well below the reported minimum inhibitory concentrations (MICs) of the corresponding ionic oxide forms. For example, in the case of 1%–10% CuFAp surfaces, approximately 0.3 mg of apatite powder was used to fabricate each disk. Assuming a worst-case scenario in which all Cu ions are leached during the bacterial assays and are fully available to impart antimicrobial activity, the maximum amount of elemental Cu would range from 0.003 μg for 1% doping to 0.03 μg for 10% doping per disc. Additionally, considering the solubility constant of CuO (2.9 × 10^−5^ mol/L [79]), the maximum amount of Cu^2+^ expected in the 50 μL inoculum used for the bacterial studies would be approximately 115 ng. At this concentration, it is unlikely that the observed antimicrobial effects of Cu-doped FAp originate from dissociated Cu^2+^ ions.

Moreover, if the antimicrobial activity were primarily driven by released metal ions, one would expect a dose-dependent increase in bacterial killing with increasing metal doping. However, neither the 10% Cu-doped FAp nor the Zn- or Ag-doped FAp samples demonstrated any statistically significant differences in the number of adhered bacteria when compared to both controls, FAp, in vitro. This observation supports that the bacteriophobic behavior is likely not governed by ionic release but may instead arise from changes in the surface properties and chemistry of FAp. In this study, all samples were sintered at 1150°C for 2 h prior to bacterial adhesion experiments to ensure structural stability during testing. Such high-temperature processing is reported to reduce the degradation properties relative to unsintered Cu-doped HAp [82]. Likewise, the sintered CuFAp surfaces may not release bacteriocidal levels of doped metal ions sufficient to elicit antimicrobial effects on the disc’s surface, providing another reason why the bacterial resistance to adhere to 1CuAps to 5CuAps should not be attributed to metal ion release. Nevertheless, it is known that sintering can induce distinct surface microstructures [83, 84], which may be further influenced by metal doping. Therefore, the observed bacteriophobic properties of the CuFAp surfaces could be attributed to the surface chemistry of the doped crystals and/or surface features generated during postsintering processing, rather than to the antimicrobial effect of metal oxides or ion release.

Interestingly, SEM micrographs (Figure 5) of the sintered, doped FAp samples revealed distinct surface topographic features that varied with both dopant type and concentration. These images also suggest that increasing dopant content was associated with an increase in grain size with Cu doping, whereas lower dopant levels resulted in smaller, more uniform grains. Such microstructural variations could be one of the reasons for differences in bacterial adhesion to these CuFAps surfaces. It is well established that micro- and nanoscale surface features can inhibit microbial fouling [85–92]. In particular, previous studies have shown that bacteria preferentially adhere to larger surface microfeatures rather than to nanoscale features [93, 94]. Collectively, this literature evidence indicates that the dimensions of surface topographic features (e.g., pillars, pores, or surface roughness) are a critical determinant of bacterial adhesion. When these features are substantially smaller than the bacterial cell size—*S. aureus* has been reported to range from approximately 0.5 to 2 μm in diameter [95]—bacteria are unable to fully conform to the surface to maximize contact area. Among the Cu-doped FAp samples, only the 10CuFAp composition exhibited relatively larger grain sizes, whereas the remaining compositions displayed smaller grains with noticeable microporosity (Figure 5). However, based on the overall surface morphology observed across all doped FAp samples, surface topography alone cannot fully explain the observed anti-adhesion behavior with 1%–5% doped-CuFAps. This finding appears to suggest that surface chemistry and charges, in addition to topography, likely play a contributory role in modulating bacterial interactions with these materials, which need further investigation.

Surface charge is known to influence the adhesion and proliferation of both prokaryotic and eukaryotic cells. The unit-cell and crystallite parameters presented in Table 3 suggest that substitution with dopant metals having smaller ionic radii (rZn^2+^ = 0.60 Å, rCu^2+^ = 0.70 Å [96]) compared with Ca^2+^ (rCa^2+^ = 1.00 Å [96]) affected the crystallite size. This size variation may contribute to surface structural changes along the c-axis, indicating that surface charge characteristics, including zeta potential, could be influenced by both the type and level of metal doping. Notably, previous studies have shown that increasing Cu dopant concentrations shifts the zeta potential from negative (0% Cu, i.e., HAp) to positive values [82]. In addition, our prior work demonstrated that FAp surfaces are negatively charged [36], consistent with other reports [82, 97]. Collectively, these findings suggest that Cu doping may alter the zeta potential of FAps surfaces, potentially contributing to their bacteriophobic properties.

It is worth noting that XRD analysis revealed that 1CuFAp and 2CuFAp exhibited a single-phase apatite structure and were successfully synthesized as pure phases, whereas 5CuFAp contained approximately 2.6% CuO. Additionally, at 2%–10% Zn doping, secondary oxide phases were observed in ZnFAp compositions. In the case of AgFAp, even at low 1% doping, the formation of elemental silver was detected. One likely explanation for oxide formation is the well-known hydrothermal reaction of metal nitrates with NaOH in the presence of metals such as Cu or Zn [98, 99]. In short, copper nitrates have been reported to react with NaOH under hydrothermal conditions (100–200°C in aqueous environments) to form copper oxide [96, 100]. Given that this synthesis process involves similar conditions, any excess metal nitrates likely reacted with NaOH (the titrant used to maintain the pH at 9.2), leading to the formation of the corresponding metal oxides. Although it is considered a limitation of the precipitation technique employed in this study to produce a higher level of Cu doping, the studied range is also cytotoxic to keratinocytes; thus, trying to improve the synthesis technique is considered beyond the scope of this study. Although these oxides were detected by XRD, our FTIR data did not show any associated oxide peaks. This lack of FTIR evidence may be due to overlapping with the characteristic P–O–P stretching and bending peaks in the same spectral region. For example, the characteristic CuO peaks at 533 and 587 cm^−1^ [101] may have been masked by the sharp PO_4_^3−^ double peaks at 571 and 602 cm^−1^ [102].

The FTIR peaks observed at around 1090 and 600 cm^−1^ for all tested metal-doped apatites indicated the presence of characteristic tetrahedral PO_4_^3−^ groups [102], and XRD data (Figure 3) further confirmed the formation of hexagonal phases. Additionally, the FTIR spectra of the raw powder did not show a sharp peak at 3565 cm^−1^, which is a known characteristic stretching band for - OH groups in HAp, thus confirming the absence of HAp as the impurities in our synthesized doped-FAp powders [103]. Therefore, within the detectable limits of FTIR, it can be concluded that the synthesis technique utilized here was able to successfully substitute hydroxyl with fluoride ions.

Although we expected to see Ag_2_O in AgFAp samples, the XRD data revealed that even at 10% Ag doping, Ag_2_O was not detected. The main impurity was elemental Ag and CaF_2_, likely resulting from the thermal treatment at 900°C prior to XRD analysis, as silver oxide—expected byproduct—is known to thermally decompose into elemental silver and oxygen above 400°C [104]. Additionally, other impurities such as CaF_2_ and secondary calcium phosphate phases, including TCP, were observed in Ag-doped samples (Figure 3). Since AgFAp did not exhibit any antimicrobial efficacy, investigating the mechanism underlying impurity formation or further optimizing the synthesis was considered unnecessary in the context of this study. In summary, the presence of elemental metals and metal oxides is therefore attributed to the synthesis method and the sintering conditions used.

The ICP-MS data revealed from Ca/P ratios calculations that powders were calcium-deficient to calcium-rich apatites (metal+Ca/P ratios of 1.48–1.93). These findings were consistent with reported values for laboratory-synthesized powders [105, 106]. Notably, the bacteriostatic 1CuFAp, 2CuFAp, and 5CuFAp samples had Ca/P ratios exceeding 1.7 (ranging from 1.73 to 1.79), classifying them as calcium-rich apatites. The ICP-MS data also revealed the Zn/Ca ratios (1.1%, 2.4%, 5.7%, and 11.9%) and Cu/Ca ratios (1.1%, 2.5%, 6.1%, and 13.8%), confirming the difference in substitution levels that were slightly higher than initially intended. Given that 10CuFAp, all Zn-doped, and all Ag-doped FAps did not exhibit bacteriostatic properties, further analysis and interpretation of these compounds were considered beyond the scope of this study.

While the 1%–5% Cu doping did produce bacteriophobic surfaces, further in vitro studies revealed a significant decrease in keratinocyte cell adhesion and viability, indicating that these surfaces lack cytocompatibility with keratinocytes. Our data revealed that, although they proliferated on undoped FAp, they displayed significant differences in adhesion and reduced growth on CuFAp samples. While copper is a well-known antimicrobial agent, it also exhibits concentration-dependent cytotoxicity to cells. However, at low levels, copper is essential for normal cell function [107–115]. Interestingly, one HAp doped study with 1–15 molar% Cu showed cytotoxicity to human fetal osteoblast cell (HFOB 1.19 cell line) [116]. Although this study used HAp, it is important to note that it was also conducted on unsintered, doped HAp discs. Because of the increase in crystallinity and the incorporation of Cu ions into the crystal structure of FAp, it was thought that the CuFAp surfaces could exhibit high cytocompatibility and therefore be a potential coating material that would improve keratinocyte adhesion, promote healing, and decrease the bacterial load. However, our cell culture data showed that the Cu doping level was perhaps too high. It is worth noting that, although only a few studies have investigated Cu-doped apatites, studies on nano copper particles showed that, at low concentrations, copper can exhibit protective cell functions [107, 109, 114]. Thus, we are currently carrying out in vitro testing with lower copper doping levels (0.25–1.0 CuFAps), as predicted, the data did show cytocompatibility and is currently accepted for publication.

This study has several limitations. While we successfully demonstrated the bacteriostatic effects of 1%–5% Cu doping against *S. aureus*, other clinically relevant pathogenic bacteria were not evaluated, highlighting the need for further investigation using 1–5CuFAps. The 2-h exposure period may also be insufficient to fully assess the material’s resistance to bacterial adhesion; longer-term studies are necessary to determine whether the surfaces are bactericidal and capable of preventing biofilm formation. Carbonate formation, an inherent issue with the precipitation-based FAp synthesis method, represents another limitation. Previous reports suggest that alternative approaches, such as sol–gel or hydrothermal synthesis under inert atmospheres, can minimize carbonate incorporation and yield stoichiometrically pure FAp [116–119]. Although zeta potentials were not measured in this study, dopant concentration likely influences surface charges, which in turn affect bacterial adhesion. Future work should incorporate zeta potential analyses to better interpret the bacteriostatic behavior of metal-doped FAps. A key limitation of this study is the absence of cytotoxicity data for the doped-FAps included in this study. Future studies will address this gap by conducting ISO 10993–5–compliant cytotoxicity testing through an independent, certified laboratory as part of comprehensive preclinical validation. As part of ongoing efforts to develop copper-doped, bacteriophobic yet bioactive FAp bone scaffolds, we also plan to examine the effects of varying sintering temperatures, which have been shown to modulate surface topography [120, 121].

## 5 | Conclusions

Characterization confirmed the successful synthesis of FAp with no detectable HAp form and effective incorporation of the dopant metals into the FAp lattice structure. While ZnFAp and AgFA did not consistently exhibit bacteriostatic activity against *S. aureus*, CuFA at 1%, 2%, and 5% demonstrated significant bacteriostatic effects, achieving approximately 3-log fold reduction in bacterial counts compared to undoped FAp. However, these copper-doping levels did not show cytocompatibility to HaCaT cells. These findings suggest that lower than 1CuFA should be explored to achieve both bacteriophobic and cytocompatibility surfaces for percutaneous OI device application.

## Figures and Tables

**TABLE 1 |:** A table showing the calcium nitrate and metal nitrates concentrations (molarities) used for synthesizing metal-doped FAps.

Percent metal (Mol%)	Ca(NO_3_)_2_	Zn(NO_3_)_2_, Cu(NO_3_)_2_, OR Ag(NO_3_)
0	2.400 M	0.0 M
1	2.376 M	0.024 M
2	2.352 M	0.048 M
5	2.280 M	0.120 M
10	2.160 M	0.240 M

**TABLE 2 |:** A table showing the calculated molar ratios of dopant/Ca and (dopant + Ca)/P for doped apatites using ICP-MS data.

Material ID	Molar ratio	1% doped	2% doped	5% doped	10% doped
ZnFAp	Zn:Ca	0.011:1	0.024:1	0.057:1	0.119:1
	(Zn+Ca):P	1.56:1	1.48:1	1.61:1	1.93:1
CuFAp	Cu:Ca	0.011:1	0.025:1	0.061:1	0.138:1
	(Cu+Ca):P	1.79:1	1.73:1	1.73:1	1.70:1
AgFAp	Ag:Ca	0.015:1	0.025:1	0.084:1	0.195:1
	(Ag+Ca):P	1.56:1	1.60:1	1.68:1	1.67:1

**TABLE 3 |:** A table showing the lattice parameters calculated using XRD data for all investigated metal-doped apatites.

Material	*a* (Å)	*c* (Å)	Material	*a* (Å)	*c* (Å)	Material	*a* (Å)	*c* (Å)
FAp	9.375	6.887						
1ZnFAp	9.368	6.877	1CuFAp	9.367	6.883	1AgFAp	9.376	6.884
2ZnFAp	9.362	6.871	2CuFAp	9.362	6.881	2AgFAp	9.372	6.891
5ZnFAp	9.359	6.868	5CuFAp	9.364	6.875	5AgFAp	9.377	6.888
10ZnFAp	9.361	6.852	10CuFAp	9.358	6.872	10AgFAp	9.382	6.892

**TABLE 4 |:** A table showing the percentages of oxides (ZnO, CuO) and elemental Ag detected within the synthesized powders (calculated based on XRD data; Figure 3), along with reported minimum inhibitory concentrations (MICs) and solubility constants of respective metal oxides.

Material	1%-doping	2%-doping	5%-doping	10%-doping	MIC of metal oxide	Solubility constant of metal oxide
ZnO	—	1.4%	2.4%	4.1%	2.5–5 mM [73]	7.4 mg/L [74]
CuO	—	—	2.1%	5.8%	100 μg/mL–2.5 mg/mL [75–78]	2.9 × 10^−5^ mol/L [79]
Ag	0.4%	0.7%	0.8%	1.4%	0.84–1.69 mg/L [80]	N/A

## Data Availability

The data that support the findings of this study are available from the corresponding author upon reasonable request.
